# Screening, Safety Evaluation, and Mechanism of Two *Lactobacillus fermentum* Strains in Reducing the Translocation of *Staphylococcus aureus* in the Caco-2 Monolayer Model

**DOI:** 10.3389/fmicb.2020.566473

**Published:** 2020-09-16

**Authors:** Zhen Peng, Benliang Wei, Tao Huang, Zhanggen Liu, Qianqian Guan, Mingyong Xie, Haijuan Li, Tao Xiong

**Affiliations:** ^1^School of Food Science and Technology, Nanchang University, Nanchang, China; ^2^State Key Laboratory of Food Science and Technology, Nanchang University, Nanchang, China; ^3^College of Biological and Environmental Engineering, Xi’an University, Xi’an, China

**Keywords:** lactobacilli, *Staphylococcus aureus*, translocation, inhibition, intestial epithelial cells

## Abstract

*Staphylococcus aureus* is a common commensal of humans, and its translocation from gastrointestine to peripheral organs and tissues could cause severe diseases and complications. This study focuses on the screening and characterization of *Lactobacillus* strains with significant inhibitory effect on the translocation of *S. aureus* through Caco-2 monolayers. First, strains with strong affinity for mucin and Caco-2 cells were obtained, via microtiter plate assay and adhesion assay, respectively. Obtained bacteria were further tested for their inhibitory effects on the growth of *S. aureus* by well diffusion assay. Subsequently, two strains preincubated with Caco-2 monolayers were found to inhibit the translocation of *S. aureus* CMCC26003 by 80.95 and 43.96%, respectively, via the transcellular translocation assay. These two strains were then identified to be *Lactobacillus fermentum* NCU3087 and *L. fermentum* NCU3088. Second, the mechanism of inhibition was investigated by analyzing the relative concentration of tight junction proteins and proinflammatory cytokines of Caco-2 cells, by Western blot and enzyme-linked immunosorbent assay, respectively. Results showed that both NCU3087 and NCU3088 significantly attenuated the degradation of occludin, claudin-1, ZO-1, and JAM-1 and suppressed the secretion of interleukin 6 and tumor necrosis factor-α induced by *S. aureus*, to different extent. Moreover, two *Lactobacillus* strains could barely translocate the Caco-2 monolayers, had no hemolytic activity, displayed strong resistance to gastrointestinal fluids, and were sensitive or moderate sensitive to nine clinically relevant antibiotics. Collectively, this study identified two *Lactobacillus* strains with significant inhibitory effect on the translocation of *S. aureus*, and their safeness for application was evaluated, thereby providing potential solutions for prevention of *S. aureus* and prophylaxis of related diseases.

## Introduction

*Staphylococcus aureus* is a common commensal of human skins and mucosal membranes (Argudín et al., 2010). The spectrum of staphylococcal infections spans, largely depending on numerous virulence factors. As 30–50% humans are *S. aureus* carriers, food handlers carrying this bacterium on the hands or in the noses are regarded as the main source of food contamination (Loir et al., 2009). Consumption of foods contaminated with sufficient amounts of preformed enterotoxin can lead to staphylococcal food poisoning. Therefore, *S. aureus* is a leading cause of gastroenteritis (Hennekinne et al., 2012). Moreover, *S. aureus* present in gastrointestine is a potential threat to public health, as it can cause endocarditis, bacteremia, pneumonia, osteomyelitis, cellulitis, and septic shock by translocation to the peripheric organs and tissues of people with a weakened immune system such as the elderly and pregnant women (Jensen et al., 2013; Tong et al., 2015; Di Cerbo et al., 2016). *S. aureus* has long been notorious for its pathogenicity and refractory, while there is no effective vaccine. Antibiotics as the current therapeutic approach have been reported to be less effective due to bacterial adaption and resistance (Jiang et al., 2019). Therefore, alternative strategies are urgently needed for the prevention of *S. aureus*.

A variety of probiotics were reported to display inhibitory effect on the adhesion, invasion, and translocation of pathogens (Gill et al., 2001; Quan Shu, 2001; Fukuda et al., 2011; Yang et al., 2014), despite the rare cases of diarrhea and acute gastroenteritis for vulnerable populations such as immunocompromised individuals (Di Cerbo et al., 2016). One important mechanism of probiotics reducing pathogen infection is adhesion abrogation by their strong affinity for host receptors such as mucin and epithelium cells (Alemka et al., 2010). Other mechanisms are speculated to be innate secretion of antagonistic substances, promotion of host immunocompetence, enhancement of intestinal barrier, etc. (Fukuda et al., 2011; Yang et al., 2014). Such lactic acid bacteria (LAB) strains have the potential to reduce or even replace the use of antibiotics in clinical or in livestock and poultry industries. The advantages of LAB replacing antibiotics such as easy to obtain, no resistance, and pollution-free attract more and more researchers to seek strains with inhibitory effects on the infection of pathogens. However, application of stochastic LAB strains to prevention of pathogens may fail due to the strain-specific property (Navasa et al., 2002; Òdena et al., 2009; McNaught et al., 2015). The underlying inhibitory effect of LAB on the adhesion and translocation of *S. aureus* needs to be excavated through elaborated screening procedure.

Consequently, a four-step screening procedure was established in this study to screen LAB strains with potential ability to inhibit translocation of *S. aureus* CMCC26003 through Caco-2 monolayers. First, mucin as the inherent substance on the surface of epithelia was used to screen LAB strains that can withstand rinsing by the fluids in the gastrointestinal environment, via microtiter plate assay. Second, Caco-2 monolayers were used to further probe LAB strains that can remarkably adhere to epithelial cells. Thirdly, well diffusion assay was proceeded to select LAB strains with inhibitory effect on the growth of *S. aureus*. Finally, two *Lactobacillus fermentum* strains that displayed significant inhibitory effect on the translocation of *S. aureus* were obtained through the transcellular translocation assay. Thereafter, the mechanisms of inhibition were investigated by analyzing the changes of tight junction proteins and inflammatory cytokines level. Moreover, lactobacilli safeness and resistance, such as bacterial translocation, hemolytic activity, regulation of virulence genes, survival in the gastrointestinal environments, and resistance to various antibiotics, were evaluated to confirm their applicability in food and pharmaceutical industry (Vesterlund et al., 2005; da Cunha et al., 2012; Songisepp et al., 2012; Nishiyama et al., 2016). This study identified two *L. fermentum* strains with significant inhibitory effect on the translocation of *S. aureus*, thereby providing potential solutions for control of *S. aureus* and prophylaxis of related diseases.

## Materials and Methods

### Bacterial Strains

In our former study, 105 LAB strains (15 *Pediococcus* and 90 *Lactobacillus*) were isolated from tradition Chinese sauerkraut (Liu et al., 2019). All isolates with an inoculum size of 5% were grown at 37°C in De Man, Rogosa, and Sharpe (MRS) broth (Solarbio, Beijing, China) without aeration for 20 h. *S. aureus* CMCC26003 was purchased from Guangdong microbial culture collection center and cultured at 37°C with constant shaking at 150 revolutions per minute (rpm) in Luria–Bertani (LB) broth (Solarbio, Beijing, China) on a shaking incubator. For plate count assay, the liquid medium was added with 1% (wt/vol) agar.

### Screening of LAB With Significant Affinity for Mucin

This study adopted microtiter plate assay to evaluate the mucin-attaching ability of 105 LAB strains according to a previously reported method (Vesterlund et al., 2005). Briefly, 150 μL mucin solution (20 mg/mL, swine source, Sigma–Aldrich, St. Louis, MO, United States) was immobilized onto 96-well plate (Corning Costar, Cambridge, MA, United States) by incubation at 37°C for 90 min then at 4°C overnight. The unbound sites were blocked by incubation with 1% BSA in phosphate-buffered saline (PBS) for 1 h at 37°C, and then plates were washed three times with PBS. LAB strains washed by PBS and resuspended in PBS with an OD_570_ of 0.5 was added into each well and incubated at 37°C for 1 h. Plates were washed three times to remove unbound bacteria, fixed by formalin solution (4%, vol/vol) for 20 min at 50°C. Then crystal violet (0.5%, wt/vol) was added to plates and incubated at 37°C for 45 min. Crystal violet was then dissolved by citrate buffer (20 mmol/L, pH 4.3) at room temperature for 45 min, and Abs_570_ was measured on a microtiter plate reader. One blocked well without bacteria was maintained as the negative control. Experiments were performed independently in triplicate, and the results are presented as an average of three replications.

### Screening of LAB With Significant Affinity for Caco-2 Cells

Caco-2 cells were cultured in Dulbecco’s modified Eagle’s medium (DMEM) (Solarbio, Beijing, China) supplemented with 10% fetal bovine serum (Clark Bioscience, Virginia, United States) and 1% penicillin-streptomycin solution in an incubator with a 5% CO_2_ atmosphere at 37°C. Above screened LAB strains were tested for their adhesion to Caco-2 cells according to our previously reported method with modifications (Peng et al., 2014). The Caco-2 cells were incubated in Transwell Permeable Supports (1.12 cm^2^, 3 μm pore size, 12 well, Corning Costar, Cambridge, MA, United States) with an initial concentration of 1 × 10^5^ cells/well. The chamber contained 0.5 mL and 1.5 mL DMEM in the apical and basolateral compartment, respectively. The DMEM medium was changed every second day for 7 days and then changed every day until confluent (approximately 3 × 10^5^ cells/well). The monolayer was shaped after growing for 16 days to differentiate and form tight junctions, and the integrity of the monolayer was evaluated by measuring the transepithelial electrical resistance (TEER) using an Millicell ERS-2-volt ohm meter (Millipore, Bedford, MA, United States) coupled to an Endohm chamber, and only values >500 Ω⋅cm^2^ were accepted for use (Ko et al., 2007; Beloqui et al., 2017; Daisley et al., 2019). LAB strains were cultured overnight and harvested by centrifugation (10,000 *g*, 10 min, 4°C), then resuspended in PBS at a concentration of about 6 × 10^7^ colony-forming units (CFU)/mL. To reach a MOI of 100, 0.5 mL LAB were added onto Caco-2 monolayer. LAB strains and Caco-2 monolayer were cocultured at 37°C under 5% CO_2_ atmosphere. After 2 h of incubation, Caco-2 monolayer were washed three times with PBS to remove unbound bacteria. Caco-2 cells and attached bacteria were then detached using 0.05% trypsin-EDTA solution and then plated on an MRS agar plate by serial dilution. Colonies were counted after 24–48 h of incubation at 37°C. The adhered LAB number per Caco-2 cell was calculated. Experiments were performed independently in triplicate, and the results are presented as an average of three replications ± SD.

### Inhibitory Effect of LAB on *S. aureus* Growth

Above screened two LAB strains with significant adherence to mucin and Caco-2 cells were further used to evaluate their inhibitory effects on the growth of *S. aureus*. Two LAB strains were cultured overnight, and the fermentation supernatants with different end point pH (pH 4.0 and 4.2) were harvested. An aliquot of each supernatants was treated with sodium hydroxide solution (adjust pH to 6.2), heat (121°C, 20 min), catalase (1 mg/mL, final concentration), papain (200 μg/mL, final concentration), proteinase K (200 μg/mL, final concentration), trypsase (200 μg/mL, final concentration), or pepsase (200 μg/mL, final concentration). MRS medium with different pH value (pH 6.2, 4.2, 4.0) was used as the control. The antibacterial activity of LAB culture broth was evaluated by well diffusion assay (Venkadesan et al., 2015). *S. aureus* CMCC26003 was handled with biosafety level two facilities and practices were complied with the biosafety regulation ZHCADC-PGC09-2010. Petri dishes containing 20 mL of media with agar were prepared previously and inoculated with 0.1 ml of 24-h broth culture of pathogenic bacteria. Once solidified the dishes were stored for 2 h in a refrigerator. For each LAB strain, three wells were slotted and filled with equal volume of above-mentioned LAB supernatants or MRS medium, and the Petri dishes were incubated at 37°C for 24 h. Then the diameter of the inhibition zone was measured with Vernier caliper in mm. Experiments were performed independently in triplicate, and the results are presented as an average of three replications ± SD.

### Inhibitory Effect of LAB on the Translocation of *S. aureus*

Evaluation of bacterial translocation was performed as described previously (Peng et al., 2014). Experiments were divided into two groups: I, Caco-2 monolayers were preincubated with LAB before *S. aureus* infection. II, Caco-2 monolayers were infected with *S. aureus* and then postincubated with LAB. For group I, Caco-2 monolayer were washed three times with antibiotic-free DMEM before infection. To achieve a multiplicity of infection (MOI) of 100, approximately 0.5 mL of LAB in DMEM (6 × 10^7^ CFU/mL) was added to the apical chamber, and the basolateral chamber was filled with 1.5 mL DMEM. The plate was centrifuged (600 *g*, 15 min) to enhance bacterial attachment to Caco-2 cells and then incubated for 2 h. Subsequently, unbound bacterial cells were washed away by PBS, the apical chamber was filled with 0.5 mL of pathogenic bacteria in DMEM (3 × 10^7^ CFU/mL), basolateral chamber was filled with 1.5 mL DMEM and incubated for another 2 h. Wells without LAB preincubation was used as the control. For group II, all conditions remained the same except the order of added bacteria reversed. Bacteria in the basolateral chambers were counted by serial dilution on LB agar plates. Experiments were performed independently in triplicate, and the results are presented as an average of three replications.

### Level of Tight Junction Proteins Regulated by LAB Strains

Level of tight junction proteins and trans-epithelial electric resistance of Caco-2 cells were determined to evaluate the barrier integrity. To measure the level of tight junction proteins, Caco-2 cells were designated to four groups as mentioned above. Western blot was used to analyze the relative quantity of occludin, claudin-1, JAM-1, and ZO-1 using β-actin as the internal control. The Caco-2 cells in the four groups were washed with cold PBS for three times and homogenized in 150 μL chilled radioimmunoprecipitation assay buffer, including protease and phosphatase inhibitors. The homogenized samples were centrifuged at 10,000 *g* for 5 min at 4°C and supernatant was collected. The concentration of protein was determined by the bicinchoninic acid assay (Solarbio Science & Technology Co., Ltd., Beijing, China). All samples were diluted with 5 × SDS loading buffer and denatured at 100°Cfor 10 min. Equal amounts of proteins (20 μg) were electrophoresed on SDS-polyacrylamide gels and electro-transferred to polyvinylidene difluoride membrane (Millipore, United States). The membranes were blocked for 2 h at room temperature with 5% skim milk (dissolved in PBS + 0.05% Tween 20, PBST) and incubated with primary antibody occludin (Abcam, Cambridge, United Kingdom), claudin-1 (Abcam, Cambridge, United Kingdom), JAM-1 (Abcam, Cambridge, United Kingdom), ZO-1 (Proteintech, Wuhan, China), and β-actin (Bioss, Beijing, China). After this, membranes were washed three times and incubated with horseradish peroxidase–coupled secondary antibody (Beyotime Institute of Biotechnology, Shanghai, China). The results were observed and analyzed using an enhanced chemiluminescence reagent kit (Beyotime Institute of Biotechnology, Shanghai, China) and Tanon-6200 gel imaging and analysis software. Meanwhile, TEER of Caco-2 monolayers was measured at 2 h. Experiments were performed in triplicate, and the results are presented as an average of three replications ± SD.

### Secretion of Cytokines of Caco-2 Cells Regulated by LAB Strains

The differentiated Caco-2 monolayers grown in Transwell plates were allocated into four groups (Zeng et al., 2017): I, Caco-2 monolayer without treatment (control). II, Caco-2 monolayer incubated with LAB strains (10^8^ CFU/mL) for 2 h. III, Caco-2 monolayer incubated with *S. aureus* (10^6^ CFU/mL) for 2 h. IV, Caco-2 monolayer preincubated with LAB (10^8^ CFU/mL) for 2 h and postinfected by *S. aureus* (10^6^ CFU/mL) for 2 h. The levels of interleukin 6 (IL-6), IL-8, IL-1β, and tumor necrosis factor-α (TNF-α) were determined as follows (Boonma et al., 2014): Culture medium was collected and centrifuged for 10 min to pellet residual bacteria, and the supernatant was used for determination of proinflammatory cytokines concentration by using an enzyme-linked immunosorbent assay (ELISA) kit (BOSTER Biological Technology Co. Ltd., Wuhan, China). Experiments were performed in triplicate, and the results are presented as an average of three replications ± SD.

### Survival of LAB in Simulated Gastrointestinal Environments

To further evaluate the survival of selected LAB strains in the harsh environment of human gastrointestine, simulated gastric and intestinal juices were prepared (Archer and Halami, 2015). Artificial gastric fluid (pH 3.0) is composed of NaCl (0.72 g/L), KCl (0.05 g/L), NaHCO_3_ (g/L), and pepsin (0.3 g/L). The intestinal juice (pH 8.0) is composed of pancreatin (0.1%, wt/vol) and bile salts (0.3%, wt/vol). The pepsin and pancreatin solutions were prepared fresh and sterilized via a 0.22 μm filter membrane. Overnight cultured LAB cells were centrifuged (6,000 *g*, 5 min, 4°C) and re-suspended in 0.9% (wt/vol) saline buffer, followed by inoculation into the gastric fluid (final cell number: 10^8^ CFU/mL), and incubated at 37°C for 3 h. Samples were taken every hour to determine survival rate by plate count method. Subsequently, 1 mL of the culture from gastric fluid was transferred to 9 mL of simulated intestinal juice and incubated at 37°C for 8 h. Samples were taken at different time point to determine the survival rate by plate count method. In this study, survival rate = Nt/N_0_ × 100%, N_*t*_ is the total viable count of LAB after treatment with simulated GI juices and N_0_ represents the total initial number of LAB. Experiments were performed in triplicate, and the results are presented as an average of three replications ± SD.

### Antibiotic Susceptibility Assay

LAB strains were tested for resistance against 15 clinically relevant antibiotics [penicillin (10 μg), amoxicillin (10 μg), cefotaxime (30 μg), bencicillin (10 μg), vancomycin (30 μg), gentamicin (10 μg), kanamycin (30 μg), streptomycin (10 μg), tetracycline (30 μg), erythromycin (15 μg), norfloxacin (10 μg), ciprofloxacin (5 μg), rifampic (5 μg), chloramphenicol (30 μg), clindamycin (2 μg)] by the Kirby-Bauer test as described by da Cunha (da Cunha et al., 2012). The antibiotic discs were purchased from Microbial Reagent Co., Ltd. (Guangdong Province, China). Diameters of the inhibition halos were measured using Vernier caliper in mm and the resistance to each antibiotic was graded according to supplier’s specifications as resistant (R), intermediate (I), or sensitive (S). The assay was performed independently in three trials, and the results are presented as an average of three replications ± SD.

### Translocation of Lactobacilli Through the Caco-2 Cell Monolayer

Translocation of the selected LAB strains was conducted according to the method of *S. aureus* translocation. The multiplicity of infection (MOI) was set to 100, and the incubation time was 2 h. Lactobacilli in the basolateral chambers were counted by serial dilution on MRS agar plates after 20 h incubation. Experiments were performed independently in triplicate, and the results are presented as an average of three replications ± SD.

### Hemolysis Test

The hemolytic activity of two LAB strains was evaluated (da Cunha et al., 2012; Songisepp et al., 2012). The pure culture was streaked onto agar plates containing 7% sheep blood, and then incubated for 48 h in anaerobic environment. *S. aureus* CMCC26003 was used as positive controls. The appearance of blood cell-solvent zone around colonies indicates the hemolysis of bacteria.

### Regulation of Virulence Genes of *S. aureus* by LAB Strains

The presence of virulence genes *nuc*, *clfA*, *FnBPA*, *Hla*, and *Hlb* in *S. aureus* was confirmed by PCR amplification. To examine whether cell–cell contact of LAB strains and *S. aureus* would induce change of virulence gene expression, relative level of virulence genes was quantified by RT-qPCR (Yang et al., 2014). Briefly, 1.5 × 10^8^ CFU/mL *S. aureus* was incubated at 37°C for 4 h with 1.5 × 10^8^ CFU/mL of *L. fermentum* NCU3087 and *L. fermentum* NCU3088, respectively. *S. aureus* without any treatment was used as the control. Total RNA was extracted from bacteria by helicase digestion and RNAprep pure Cell/Bacteria Kit (TIANGEN, Beijing, China) according to manufacturer’s manual. RNA was further purified and treated with DNase I on QIAGEN RNeasy Midi columns (QIAGEN, Venlo, The Netherlands) according to the manufacturer’s instruction. The concentration and quality of extracted RNA were assessed by spectrophotometry at absorbance levels of 260 and 280 nm (NanoDrop 2000, Thermo Fisher Scientific, Waltham, United States). Purified RNA was suspended in RNase free ddH2O and stored at −80°C until use. Before quantitative real time PCR, RNA was reverse transcribed into cDNA with PrimeScript^TM^ RT reagent Kit with gDNA Eraser (Takara, Japan) according to the manufacturer’s protocol and stored at −20°C until use. Obtained cDNA was used as template in qPCR to determine the expression of *nuc*, *clfA*, *FnBPA*, *Hla*, and *Hlb*. *16s rRNA* was used as the internal control (housekeeping gene). Primers for virulence genes were designed and synthesized (Sangon Biotech, Shanghai Co., Ltd.) according to previous reports (Table 1; Ster et al., 2005; Karmakar et al., 2018). Real-time PCR was performed using the above prepared DNA intercalating fluorescent agent SYBR green for product detection. The PCR mixture contained 12.5 μL of SYBR Premix Ex Tap II (2×) (PE Applied Biosystems), 2 μL of cDNA at 10 ng/ml, and 0.4 μM concentrations of each primer in a 25 μL final volume. Real-time PCR amplification was initiated at 95°C for 30 s, followed by 40 cycles of 95°C for 10 s and 55°C for 30 s. Relative expression of genes was calculated using the delta-delta Ct method.

**TABLE 1:** Primers for virulence gene of *S. aureus*.

Gene	Primer sequence	Product length (bp)
*16s rRNA*	Forward (5′–3′): TATGGAGGAACACCAGTGGCGAAG	116
	Reverse (5′–3′): TCATCGTTTACGGCGTGGACTACC	
*clfA*	Forward (5′–3′): TGCAACTACGGAAGAAACGCCG	104
	Reverse (5′–3′): CCTCCGCATTTGTATTGCTTGATTG	
*FnBPA*	Forward (5′–3′): CGACACAACCTCAAGACAATAGCGG	133
	Reverse (5′–3′): CGTGGCTTACTTTCTGATGCCGTTC	
*hla*	Forward (5′–3′): GCGAAGAAGGTGCTAACAAAAGTGG	195
	Reverse (5′–3′): CGCCAATTTTTCCTGTATCATCACC	
*hlb*	Forward (5′–3′): CGACCGTTTTGTATCCAAACTGGG	200
	Reverse (5′–3′): TTTGTCCCACCCTGATTGAGAACG	
*nuc*	Forward (5′–3′): GCGATTGATGGTGATACGGTT	270
	Reverse (5′–3′): AGCCAAGCCTTGACGAACTAAAGC	

### Statistical Analysis

Data analyses were performed by using GraphPad Prism version 5.02 for Windows (GraphPad software, San Diego, CA). Statistical significance (*P* < 0.05) was evaluated by one-way analysis of variance (ANOVA). This study uses different letters (a, b, c, d, e, f, g) to show statistically significant differences between variables. If two variables have the same letters, they are not significantly different.

## Results

### Screening of LAB With Strong Affinity for Mucin and Caco-2 Cells Inhibiting *S. aureus* Growth and Translocation

**FIGURE 1:**
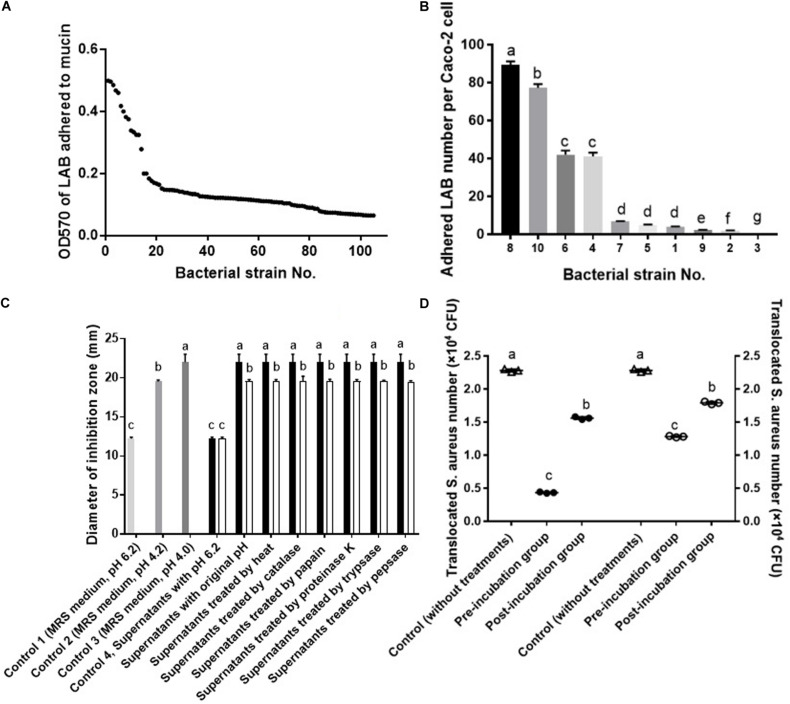
Screening of LAB with strong affinity for mucin and Caco-2 cells inhibiting *S. aureus* growth and translocation. **(A)** Adhesion of LAB to mucin by microtiter assay. **(B)** Adhesion of LAB to Caco-2 cells by adhesion assay. **(C)** Inhibitory effect of LAB fermentation supernatants on the growth of *S. aureus* by well diffusion assay. ■ supernatants of NCU3087, □ supernatants of NCU3088. **(D)** Inhibitory effect of LAB on the translocation of *S. aureus* by the transcellular translocation assay. △ Control (without treatment of lactobacilli), ∙ treated with NCU3087, ₒ treated with NCU3088. The experiments were performed in triplicate and the data is mean (±SD) except for **(A)**. Different letters indicate statistically significant differences between variables.

A total of 105 LAB strains were evaluated for their adhesion ability to mucin via microtiter plate assay. As shown in Figure 1A, bacteria showed remarkably different ability to bind mucin, of them, strains with an OD_570_ of more than 0.4 (*P* < 0.05) were further used to select strains with strong adherence to Caco-2 cells. As shown in Figure 1B, adhesion of bacteria to Caco-2 cells was strain-dependent. Of them, strain No.8 displayed the strongest adhesion to Caco-2 cells, followed by strain No. 10. The top two (*P* < 0.05) bacterial strains (No. 10 and No. 8) with strong affinity for Caco-2 cells were further tested for their ability to inhibit *S. aureus* growth. Bacteria No. 10 and No. 8 were previously identified (Liu et al., 2019) and confirmed in this study to be both *L. fermentum* strains by 16s rRNA sequencing, and then named *L. fermentum* NCU3087 and *L. fermentum* NCU3088, respectively. As shown in Figure 1C, both the fermentation supernatants of *L. fermentum* NCU3087 (pH 4.0) and *L. fermentum* NCU3088 (pH 4.2) displayed inhibitory effect on pathogen growth and this effect was not affected by the treatments of heat, catalase, papain, proteinase K, trypsase and pepsase, but disappeared after treatment with sodium hydroxide to adjust pH to 6.2. Thereafter, *L. fermentum* NCU3087 and *L. fermentum* NCU3088 were used to evaluate the ability to inhibit *S. aureus* translocation. As shown in Figure 1D, in comparison with the group without any treatments (control group), translocation of *S. aureus* significantly reduced in the groups treated with lactobacilli. Preincubation of Caco-2 cells with *L. fermentum* NCU3087 and *L. fermentum* NCU3088 decreased the translocation of *S. aureus* from 2.28 × 10^4^ to 4.33 × 10^3^ and 1.28 × 10^4^ (CFU/mL), respectively, corresponding to the inhibition rate of 80.95 and 43.96%, respectively. In the postincubation group, *L. fermentum* NCU3087 and *L. fermentum* NCU3088 decreased the translocation of *S. aureus* from 2.28 × 10^4^ to 1.56 × 10^4^ and 1.79 × 10^4^ (CFU/mL), respectively, corresponding to the inhibition rate of 31.52 and 21.21%, respectively.

### Influence of Lactobacilli on Epithelial Permeability

**FIGURE 2:**
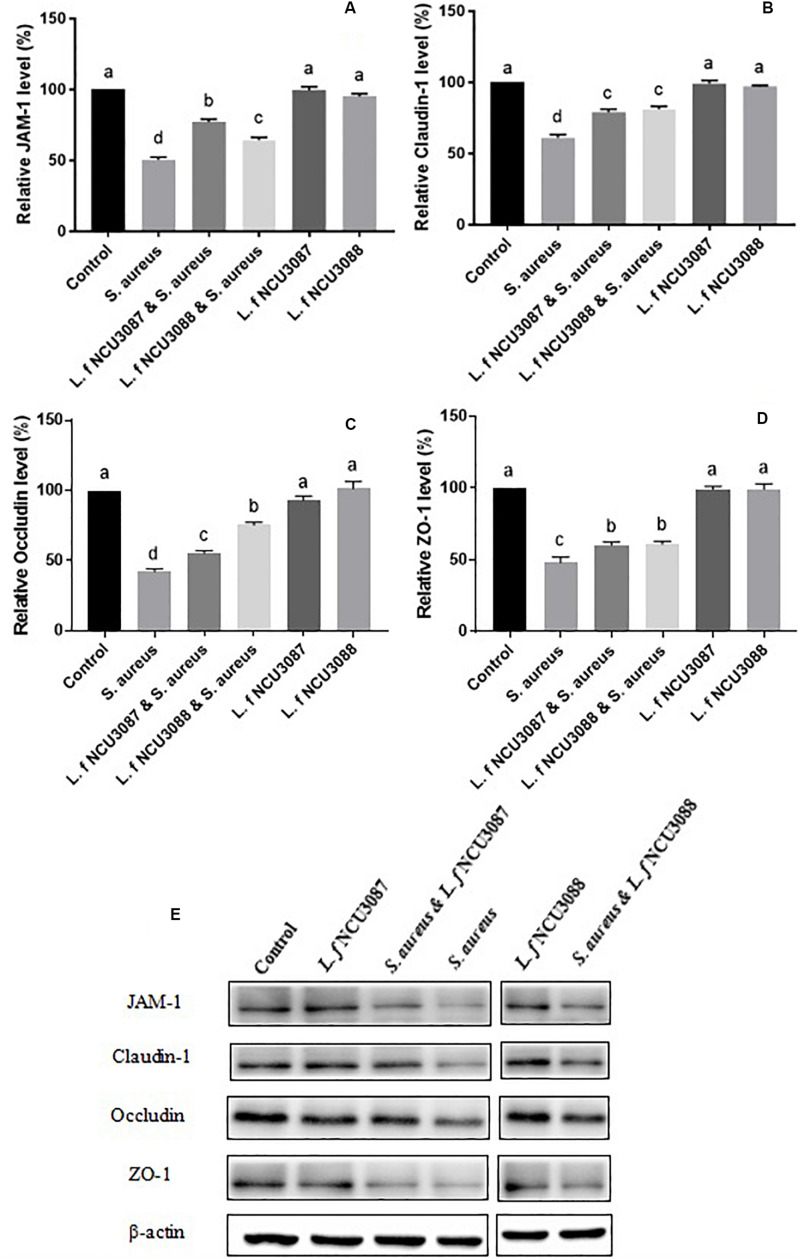
Quantification of JAM-1 **(A)**, Claudin-1 **(B)**, Occludin **(C)**, and ZO-1 **(D)** of Caco-2 cells treated by bacteria via Western blot analysis **(E)**. Different letters indicate statistically significant differences between variables.

**FIGURE 3:**
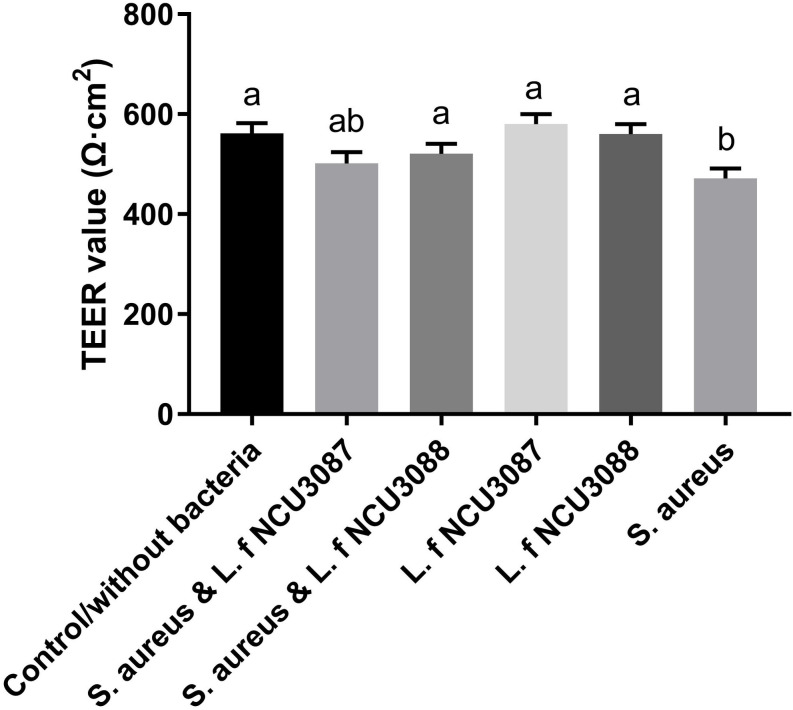
TEER level of Caco-2 monolayer after incubation with bacteria. The experiments were performed in triplicate, and the data are mean ± SD. Different letters indicate statistically significant differences between variables.

To find out whether selected *L. fermentum* strains inhibited the translocation of *S. aureus* by regulation of intestinal barrier permeability, the level of tight-junction proteins of Caco-2 cells was determined. As shown in Figure 2, infection of *S. aureus* significantly degraded occludin, claudin-1, ZO-1 and JAM-1, and preincubation with *L. fermentum* NCU3087 or *L. fermentum* NCU3088 significantly attenuated degradation of these tight junction proteins, but still had a significant difference with the control group. Besides, incubation of Caco-2 cells alone with *L. fermentum* NCU3087 or *L. fermentum* NCU3088 resulted in no significant difference of tight junction protein level in comparison with the control group. Meanwhile, the decreased TEER value induced by *S. aureus* significantly elevated by preincubation with *L. fermentum* NCU3088, but not with *L. fermentum* NCU3087 (Figure 3). In addition, incubation of Caco-2 cells alone with *L. fermentum* NCU3087 or *L. fermentum* NCU3088 resulted in no significant difference of TEER level in comparison with the control group.

### Influence of Lactobacilli on Cytokines Secretion

**FIGURE 4:**
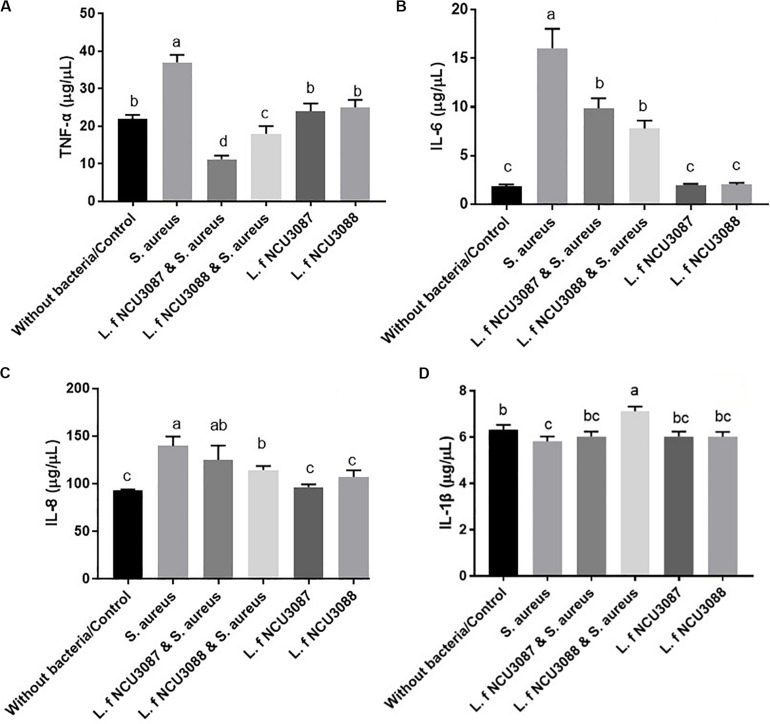
Quantification of TNF-α **(A)**, IL-6 **(B)**, IL-8 **(C)**, and IL-1β **(D)** of Caco-2 cells treated by bacteria via ELISA assay. Different letters indicate statistically significant differences between variables.

To find out if *L. fermentum* strains regulate secretion of cytokines, concentration of proinflammatory cytokines and TNF-α was determined. As shown in Figure 4, Both *L. fermentum* NCU3087 and *L. fermentum* NCU3088 significantly suppressed the secretion of TNF-α and IL-6 induced by *S. aureus*. Interestingly, *L. fermentum* NCU3088 significantly suppressed the secretion of IL-8 and promoted secretion of IL-1β induced by *S. aureus* while *L. fermentum* NCU3087 did not show significant impact. In addition, incubation of Caco-2 cells alone with *L. fermentum* NCU3087 or *L. fermentum* NCU3088 resulted in no significant difference of cytokine level in comparison with the control group.

### Survival of Lactobacilli in Simulated Gastrointestinal Environments

**FIGURE 5:**
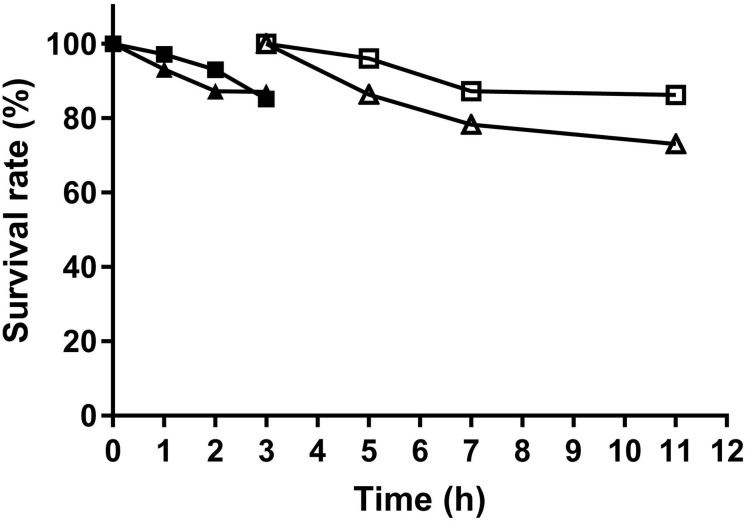
Survival of *L. fermentum* in gastric and intestinal juice. ■ NCU3087 in gastric juice, △ NCU3087 in intestinal juice, ▲ NCU3088 in gastric juice, △ NCU3088 in intestinal juice. The experiments were performed in triplicate, and the data are mean ± SD.

Two *L. fermentum* strains were tested for their tolerance to simulated gastrointestinal niches. As shown in Figure 5, cell number of survived *L. fermentum* decreased along with time. By incubation with gastric juice for 3 h, *L. fermentum* NCU3087 and *L. fermentum* NCU3088 maintained a survival rate of 85.5 and 87%, respectively. After a continuous incubation with intestinal juice for another 8 h, the survival rate for *L. fermentum* NCU3087 and *L. fermentum* NCU3088 was 86 and 73%, respectively.

### Antibiotic Susceptibility of *L. fermentum* Strains

Antibiotic resistance of *L. fermentum* NCU3087 and *L. fermentum* NCU3088 was tested by disc diffusion method. As shown in Table 2, both strains were sensitive or moderate sensitive to clinically relevant antibiotics such as cefotaxime, rifampin, chloramphenicol, ergomycin, clindamycin, amoxicillin, penicillin, tetracycline and ampicillin. To the other six tested antibiotics, both strains showed resistance.

**TABLE 2:** Antibiotic susceptibility of lactic acid bacteria.

Antibiotics	Inhibition zone (diameter in mm)		Antibiotics	Inhibition zone (diameter in mm)	
	*L.f* NCU3087	*L.f* NCU3088		*L.f* NCU3087	*L.f* NCU3088
Cefotaxime	21.20 ± 0.05 (I)	26.36 ± 0.08 (S)	Ampicillin	26.46 ± 0.07 (S)	28.46 ± 0.08 (S)
Rifampin	16.66 ± 0.02 (I)	19.38 ± 0.04 (I)	Streptomycin	− (R)	− (R)
Chloramphenicol	20.43 ± 0.04 (S)	18.01 ± 0.03 (S)	Norfloxacin	− (R)	− (R)
Ergomycin	16.39 ± 0.02 (I)	19.08 ± 0.04 (I)	Ciprofloxacin	− (R)	− (R)
Clindamycin	16.19 ± 0.02 (I)	15.96 ± 0.02 (I)	Vancomycin	− (R)	− (R)
Amoxicillin	26.58 ± 0.08 (S)	31.03 ± 0.09 (S)	Gentamicin	− (R)	− (R)
Penicillin	24.92 ± 0.07 (S)	29.13 ± 0.08 (S)	Kanamycin	− (R)	− (R)
Tetracycline	16.64 ± 0.02 (I)	19.85 ± 0.04 (I)			

*R indicates resistant, I indicates intermediate, S indicates susceptible.*

### Translocation of Lactobacilli

**FIGURE 6:**
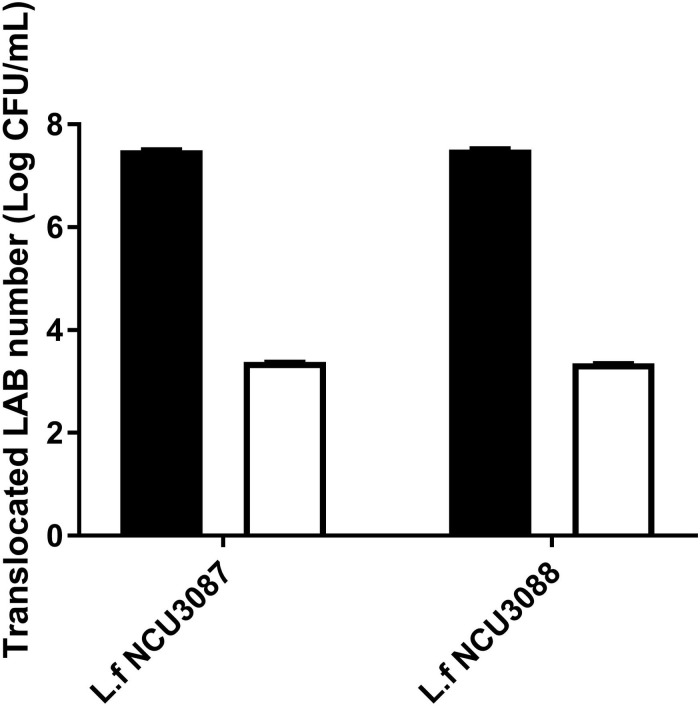
Translocation of two lactobacilli in the Caco-2 monolayer model. ■ Initial LAB number, □ translocated LAB number. The experiments were performed in triplicate, and they are mean ± SD.

Translocation of two *Lactobacillus* strains was analyzed by the transcellular translocation assay. As shown in Figure 6, with an initial infection number of around 7.5Log CFU/mL, about 3.4Log CFU/mL and 3.5Log CFU/mL of *L. fermentum* NCU3087 and *L. fermentum* NCU3088 translocated the monolayers, respectively, namely, both strains had a translocation rate of less than 0.01%.

### Hemolysis of LAB

*L. fermentum* NCU3087 and *L. fermentum* NCU3088 were grown in the presence of sheep blood to evaluate the hemolysis activity. This study observed no hemolysis for both LAB strains while positive control *S. aureus* CMCC26003 was found to lyse blood cells (data not shown).

### Regulation of Virulence Genes by Lactobacilli

Expression of virulence genes in *S. aureus* was detected by RT-qPCR, and this study set fivefold as the threshold. As shown in Table 3, preincubation with *L. fermentum* NCU3087 and *L. fermentum* NCU3088 did not affect the expression of virulence genes *nuc*, *clfA*, *Hla*, and *Hlb*, but upregulated *FnBPA* gene by 8- and 20-fold, respectively.

**TABLE 3:** Fold change of virulence genes in *S. aureus* regulated by *L. fermentum* strains via RT-qPCR assay.

*L. fermentum* strains	Fold change of virulence genes in *S. aureus*				
	*nuc*	*clfA*	*Hla*	*Hlb*	*FnBPA*
NCU3087	–	–	–	–	+8
NCU3088	–	–	–	–	+20

*This study set 5 as the threshold, ND indicates fold change is not detected.*

## Discussion

Translocation of *S. aureus* through the intestinal barrier and propagation in peripheral tissues may induce severe complications. Currently, antibiotics as the main measure to control the resultant infections is constrained due to bacterial resistance. Resistance of *S. aureus* to various antibiotics such as methicillin-resistant *S. aureus* (MRSA) had been reported. Moreover, overuse of antibiotics makes the drugs much less effective at treating infections among patients presenting to emergency departments, which is becoming a global risk especially in China, where doctors prescribe antibiotics to half of all outpatients, far above recommended levels, according to the World Health Organization (WHO). Probiotics have emerged as an alternative approach for prophylaxis of complications caused by pathogens. For example, *Bifidobacterium* (*B.*) *pseudocatenulatum* CECT7765 prevented *Escherichia* (*E.*) *coli* from migrating to the liver of mice (Moratalla et al., 2014). *B. lactis* HN019 decreased the dissemination of *E. coli* O157:H7 in mice (Quan Shu, 2001). *L. rhamnosus* HN001 reduced translocation of *Salmonella typhimurium* in mice (Gill et al., 2001). It is therefore meaningful and of importance to seek for probiotic strains with significant inhibitory effect on the translocation of *S. aureus* to hosts.

To achieve strains with significant inhibitory effect on the translocation of *S. aureus* through epithelial cells, 105 LAB strains underwent a series of screening procedure. This study revealed that lactobacilli that tend to bind with mucin do not necessarily display strong adhesion to Caco-2 cells, corresponding to the characteristic of other tested *Lactobacillus* strains (Martín et al., 2012; Garcia-Gonzalez et al., 2018). Of the 105 strains, we selected strains that displayed strong affinity for both mucin and Caco-2 cells. Subsequently, No. 10 (*L. fermentum* NCU3087) and No. 8 (*L. fermentum* NCU3088) with the strongest adhesion ability were used to investigate the inhibitory effect on the growth of *S. aureus*. Thereafter, the inhibitory effect of NCU3087 and NCU3088 on the translocation of *S. aureus* in the Caco-2 cell monolayer model was determined via the transcellular translocation assay. In comparison with the non-treated group, translocation of *S. aureus* in the group preincubated with *L. fermentum* NCU3087 and *L. fermentum* NCU3088 was inhibited by 80.95 and 43.96%, respectively. In the postincubation group, however, the inhibition rates were 31.52 and 21.21%, respectively. To the best of our knowledge, though many literatures had reported the inhibitory effect of probiotics on pathogens, yet literatures on the prevention of *S. aureus* translocation mediated by *L. fermentum* are very limited. Only Gan reported that *L. fermentum* RC-14 and its secreted biosurfactant significantly inhibited *S. aureus* infection of surgical implants in rats by adhesion abrogation (Gan et al., 2002). On the other hand, Òdena and his coworkers demonstrated that *L. fermentum* CECT 5716 had no impact on the translocation of *S. aureus* in rats (Òdena et al., 2009).

The mechanisms that *L. fermentum* strains inhibit translocation of *S. aureus* could be multiple. The strong adhesion of *L. fermentum* NCU3087 and *L. fermentum* NCU3088 to mucin and Caco-2 cells indicated that they have the potential to exclude *S. aureus* by reducing its adhesion. This was deduced by the fact that preincubation of Caco-2 monolayers with *L. f* NCU3087 or *L. f* NCU3088 resulted in a lower translocation rate of *S. aureus* comparing with the postincubation group. This is in agreement with Zhang et al., who reported that *Lactococcus lactis* excluded *S. aureus* and *E. coli* mainly by competing for adhesion sites (Zhang et al., 2015). *L. helveticus* and *L. rhamnosus* prevented the diffusion of aerobic and anaerobic bacteria into mesenteric lymph nodes of rats by abrogation of bacterial adhesion (Zareie et al., 2006). Antagonism of *S. aureus* by *L. f*ermentum strains is another mechanism counts for the inhibitory effect. This study identified no effective metabolites except for acidic substances, which displayed inhibitory effect on the growth of *S. aureus*, in that addition of catalase, papain, proteinase K, trypsase and pepsase to the supernatants did not affect the inhibitory effect (Figure 1C). This is similar to a relevant study, which reported that the lactic acid-producing bacteria inhibited *Salmonella* translocation in chickens (Yang et al., 2014). Regulation of intestinal permeability by *L. fermentum* strains was demonstrated by TEER measurement and Western blot assay. It was found that infection of *S. aureus* resulted a strong decrease of TEER value, while the group preincubated with *L. fermentum* NCU3088 displayed significantly elevated TEER value. Moreover, expression of tight junction proteins such as occludin, claudin-1, JAM-1, and ZO-1 were found to be significantly up-regulated by both *L. fermentum* NCU3087 and *L. fermentum* NCU3088. This is supported by a similar study, discovering that a LAB mixture could reduce *E. coli* K1-mediated injury of intestine by promoting expression of ZO-1 and occludin (Zeng et al., 2017). The regulatory effect of *L. fermentum* on the proinflammatory cytokines of Caco-2 cells was detected using ELISA. Level of TNF-α and IL-6 of Caco-2 cells induced by *S. aureus* was found to be significantly reduced by both *L. fermentum* NCU3087 and *L. fermentum* NCU3088, while that of IL-8 was significantly down-regulated by *L. fermentum* NCU3088 but not by *L. fermentum* NCU30887. This is similar to the regulatory effect of *L. rhamnosus* L34, which inhibited the translocation of intestinal pathogens as well as decreased IL-6 level induced by these pathogens, thereby attenuating the sepsis of murine models (Panpetch et al., 2018). In another study, VSL#3 probiotic inhibited infection of enterobacteria and enterococci and attenuated cirrhosis in rats by reducing TNF-α secretion and increasing ileal occludin level (Sánchez et al., 2015). It is worth to mention that *L. fermentum* NCU3087 or NCU3088 alone did not significantly affect the level of tight junction proteins or cytokines. However, in the presence of pathogen, the two selected LAB strains attenuated the degradation of tight junction proteins and production of cytokines induced by *S. aureus*.

To assess if *L. fermentum* NCU3087 and *L. fermentum* NCU3088 have the characteristics of probiotics for human and animal use, bacterial properties such as survival in gastrointestinal fluids, resistance to antibiotics, translocation ability and hemolytic activity were characterized. *L. fermentum* NCU3087 was found to display strong adaption to gastrointestinal fluids while *L. fermentum* NCU3088 was slightly weaker (Figure 5). Both *L. fermentum* NCU3087 and *L. fermentum* NCU3088 had a translocation rate of less than 0.01%, similar to the translocation rate of *L. casei* in a Caco-2 model (Mathipa et al., 2019). Susceptibility of *L. fermentum* NCU3087 and *L. fermentum* NCU3088 to 15 antibiotics were tested, the breakpoints used was in accordance with current European Food Safety Authority (EFSA) guidelines and the results corresponded to the antibiotic resistance among commercially available probiotics (Sharma et al., 2014). Most *Lactobacillus* species are intrinsically resistant to aminoglycosides (gentamicin, kanamycin, and streptomycin), glycopeptides such as vancomycin, and quinolones such as ciprofloxacin, and these antibiotic resistance (AR) genes are highly conserved in *Lactobacillus* species (Abriouel et al., 2015; Campedelli et al., 2019). Moreover, *L. fermentum* NCU3087 and *L. fermentum* NCU3088 displayed no hemolytic activity, confirming to their potential use in food and pharmaceuticals industry. In addition, regulation of virulence genes by *L. fermentum* was investigated via RT-qPCR method. Incubation of *S. aureus* with *L. fermentum* did not affect the expression of virulence genes *nuc*, *clfA*, *Hla*, and *Hlb*. Surprisingly, relative level of FnBPA (fibronectin binding protein A) in *S. aureus* was found to be 8- and 20-fold up-regulated by strain NCU3087 and NCU3088, respectively. This is different from *L. reuteri* RC-14, which produced molecules to suppress the expression of virulence genes such as *sarA* and *saeRS*, thereby preventing *S. aureus* infections (Di Cerbo et al., 2016). We assumed that the cell–cell contact resulted in overexpression of *FnBPA* for *S. aureus* to cope with environmental stress, i.e., coexistence with *L. fermentum* NCU3087 and *L. fermentum* NCU3088 enhanced the competitiveness of *S. aureus* in an ecological environment. This is in accordance with the study of Cameron, who reported that *Enterococcus faecalis* promoted the expression and activity of the enterohemorrhagic *Escherichia coli* Type III secretion system and enhanced its competitiveness (Cameron et al., 2019).

For future studies, there is more follow-up work to carry out. First, the model we adopted was limited to Caco-2 monolayers, which is although most frequently used to mimic the intestinal barrier, albeit could not stand for the full functions of an authentic intestine. It would be interesting to confirm the inhibitory effect of *L. fermentum* NCU3087 and *L. fermentum* NCU3088 on the translocation of *S. aureus* in an animal model. Second, because *L. fermentum* NCU3087 displayed much stronger inhibitory effect on the translocation of *S. aureus* than *L. fermentum* NCU3088, the genome information of these two strains could be excavated to find out the key genes or gene clusters that dominate the inhibitory effect.

## Conclusion

Two *L. fermentum* strains were identified for their evident inhibitory effect on the translocation of *S. aureus*. The inhibitory mechanisms probably lay in adhesion abrogation, growth inhibition, and barrier permeability improvement. The safeness such as bacterial translocation, hemolytic activity, and resistance to various antibiotics was confirmed by a series of experiments. These findings suggest that the well-characterized *L. fermentum* NCU3087 and *L. fermentum* NCU3088 have the potential to provide a novel strategy for the management of *S. aureus* and its derived complications.

## Data Availability Statement

All datasets presented in this study are included in the article/supplementary material.

## Author Contributions

ZP conceived the ideas, analyzed the data, and wrote the manuscript. BW performed the screening tests. TH performed the identification experiments. ZL performed the tolerance experiments. QG performed the virulence gene regulation experiment. MX interpreted the several results. HL critically analyzed the data. TX provided the platform and devices. All authors contributed to the article and approved the submitted version.

## Conflict of Interest

The authors declare that the research was conducted in the absence of any commercial or financial relationships that could be construed as a potential conflict of interest.
